# PReS-FINAL-2077: Lung function evaluation in a juvenile idiopathic arthritis cohort

**DOI:** 10.1186/1546-0096-11-S2-P89

**Published:** 2013-12-05

**Authors:** I Machado Vaz, M Rodrigues, I Azevedo, J Winck, I Brito

**Affiliations:** 1 Physical Medicine and Rehabilitation Department, São João Hospital, Porto, Portugal; 2 Pediatrics, São João Hospital, Porto, Portugal; 3 Pediatric Pulmonology Unit, São João Hospital, Porto, Portugal; 4 Pulmonology, São João Hospital, Porto, Portugal; 5 Pediatric Rheumatology Unit, São João Hospital, Porto, Portugal

## Introduction

Juvenile idiopathic arthritis (JIA) is the most common rheumatologic disorder of childhood. It is a group of diseases characterized by chronic synovitis, associated with many extra-articular manifestations including pulmonary involvement, usually in the systemic type.

Alterations of pulmonary function in JIA may be a complication of the underlying disease or related to treatment with methotrexate.

Numerous reports have documented the association of rheumatoid arthritis and pulmonary abnormalities. Also, methotrexate may cause severe adverse pulmonary side-effects in adults with rheumatoid arthritis.

While respiratory function in adults with rheumatoid arthritis has been extensively investigated, few studies have been carried out in JIA children.

## Objectives

To evaluate lung function in a cohort of Juvenile Idiopathic Arthritis (JIA) patients followed in a tertiary care center.

## Methods

Transversal study of consecutive JIA patients recruited in a Pediatric Rheumatology Clinic, between December 2011 and March 2012. Clinical history and past medical history were collected by using medical records and a structured questionnaire. Lung function evaluation included spirometry, carbon monoxide diffusing capacity (DLCO) and sniff nasal inspiratory pressure (SNIF). Laboratory workup included complete blood count, ESR, CRP, ANA and RF.

## Results

41 recruited patients had ages between 6 and 20 years (average 11.8 ± 4.1), 23 (62.2%) were females. 4 were lost to follow-up.

2 patients had weight <P5 and 2 with weight >P95, 1 had height <P5 and 5 with height >P95, the remaining had adequate growth.

Disease duration was 62.4 ± 29.1 months from diagnosis. 17 (45.9%) had oligoarticular, 8 (21.6%) systemic and 6 (16.2%) polyarticular type.

31(83.3%) had no active synovitis, and only 2 (5.7%) had 3 or more active joints. 60% patients were currently on medication: 10 (28.6%) methotrexate, 3 (8.6%) sulfasalazine, 1 (2.9%) anti-TNF, 1 (2.9%) anti-IL1 and 2 (5.7%) methotrexate plus anti-TNF.

No patients had previous known lung disease or respiratory symptoms. Lung function tests were mostly normal: Forced Vital Capacity (FVC%) 97.9 ± 17.0, Forced Expiratory Volume in 1 second (FEV1%) 104.2 ± 17.5, FEV1/FVC% 99.18 ± 12.2, Total Lung Capacity (TLC%) 94.6 ± 14.5, Residual Volume (RV%) 130.5 ± 53.1, DLCO% 93.1 ± 14,2, SNIF 69.6 ± 26.5. Only 3 patients had lung function changes - 2 restrictive and 1 obstructive spirometry patterns, no patients had changes in DLCO.

## Conclusion

The low prevalence of lung function changes in our cohort is in agreement with the literature. In contrast to studies performed in adult rheumatoid arthritis patients, in children with juvenile idiopathic arthritis impairment of lung function seems to be a rare event.

The small sample size, the fact that most of the patients were in remission, as well as the lack of a control group can contribute to the difficulty in interpreting the results.

Prospective follow-up of this cohort, particularly those with lung function changes, will be paramount.

## Disclosure of interest

None declared.

